# Supplementary material for: A Multi-Enzyme Cascade Response for the Colorimetric Recognition of Organophosphorus Pesticides Utilizing Core-Shell Pd@Pt Nanoparticles with High Peroxidase-like Activity
Source: Foods. 2023 Sep 4;12(17):3319. doi: 10.3390/foods12173319 (PMC10486685; doi:10.3390/foods12173319)
Supplement: Supplementary file 1 [file foods-12-03319-s001.zip › foods-2522529-supplementary.pdf]

foods

SUPPLEMENTARY FILE

A multi-enzyme cascade response for the colorimetric recognition of organophosphorus pesticides utilizing core-shell Pd@Pt nanoparticles with high peroxidase-like activity

Majid Zainabu [1], Qi Zhang [1], Zhansen Yang [1], Huilian Che [1,2,*] and Nan Cheng [1,*]

[1] Beijing Laboratory for Food Quality and Safety, College of Food Science and Nutritional Engineering, China Agricultural University, Beijing 100083, China;
[2] Key Laboratory of Precision Nutrition and Food Quality, Key Laboratory of Functional Dairy, Ministry of Education, College of Food Science and Nutritional Engineering, China Agricultural University, Beijing 100083, China
* Correspondence: chengnanfsne@cau.edu.cn

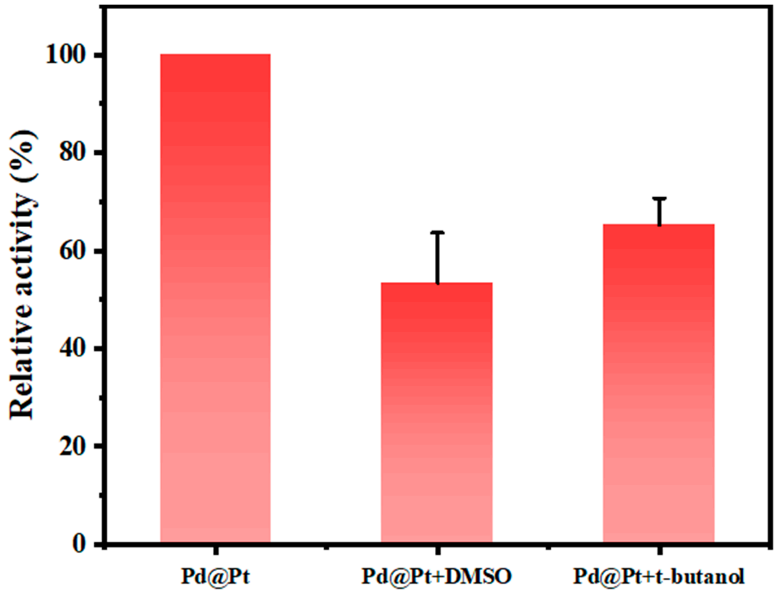

Figure S1. Effect of ·OH scavengers on the peroxidase activity of Pd@Pt. Effect of dimethyl sulfoxide (DMSO) and t-butynol on POD-like activity of Pd@Pt nanoparticles. The standard error (s.e.) was revealed as error bars after three independent experiments.

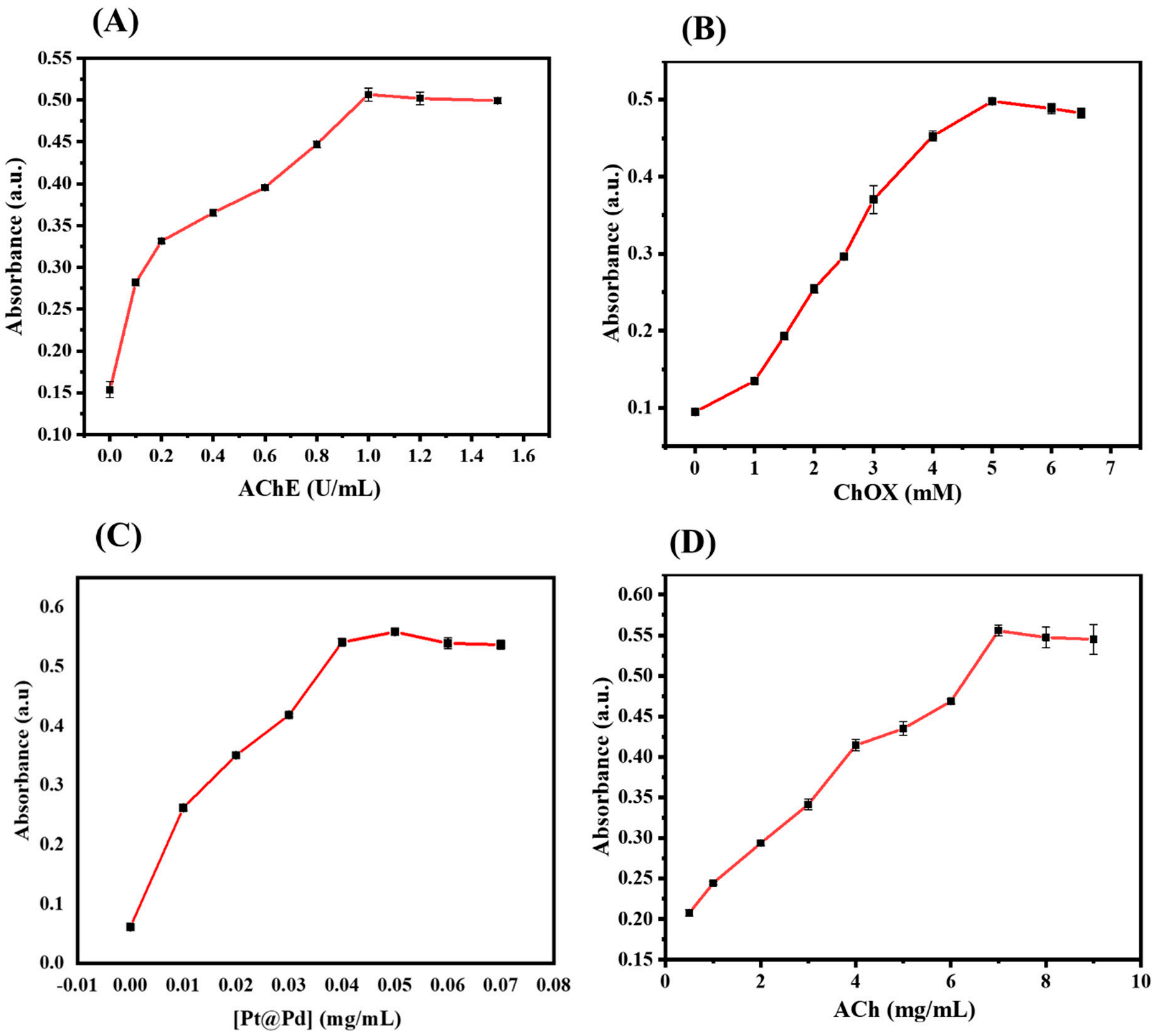

Figure S2. The condition optimization of a multi-enzyme cascade reaction. The effect of **(A)** AChE; **(B)** ChOx; **(C)** Pd@Pt nanozyme; and **(D)** ACh concentrations on different reaction system, respectively. Error bars indicate the standard error (s.e) of three independent experiments.

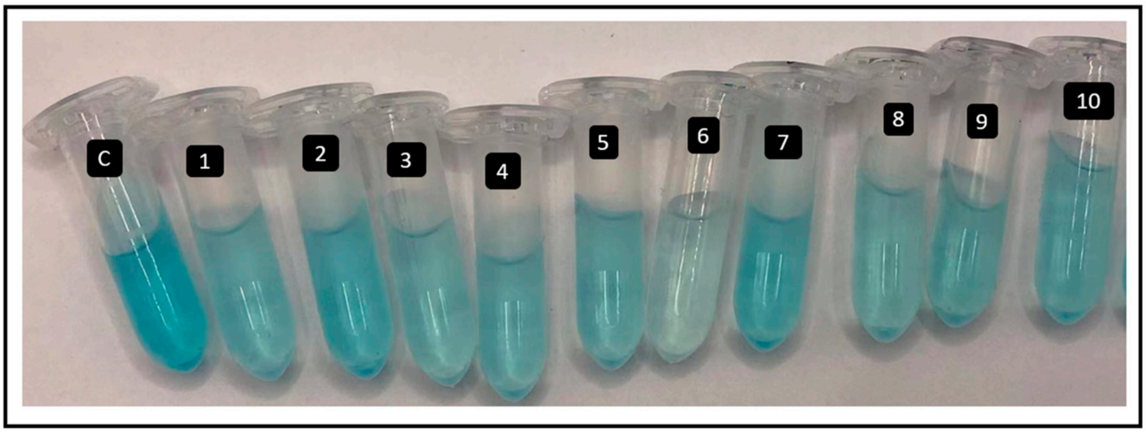

Figure S3. The representative images of real samples sprayed with Parathion. Inside are different tested samples of C-Control sample (no OP); 1-spinach; 2-apple; 3-banana; 4-cabbage; 5-brocolli; 6-tomato; 7-eggplant; 8-ginger; 9-Potatoes and 10-Mango samples. This section may be divided by subheadings. It should provide a concise and precise description of the experimental results, their interpretation, as well as the experimental conclusions that can be drawn.

POD-like activity parameters and comparison of noble metal-based nanoparticles

Table S1 POD-like activity parameters and comparison of noble metal-based nanoparticles

Category	Catalysts	K_m (mM)	V_{max} (M s^{-1})	References
Noble metal-based nanoparticles	Tyrosine-capped Ag-NPs	0.053	3.7×10^{-8}	[1]
	PtNP@UiO-66-NH2	0.127	1.36×10^{-8}	[2]
	BSA-stabilized Pt NPs	0.119	29×10^{-8}	[2]
	citrate-capped Pt NPs	0.121	6.51×10^{-8}	[2]
	Pt/cube-CeO2	0.26	1.47×10^{-7}	[3]
	Pd NCs	0.063	7.64×10^{-8}	[4]
	Au-Pt NCs	0.362	11.366	[5]
	HRP	0.4376	1.38×10^{-6}	[50]
	Pd@Pt	0.26417	0.36002	This work

K_m is the Michaelis-Menten constant, v_{max} is the maximal reaction rate

References

1. Weerathunge, P., Behera, B. K., Zihara, S., Singh, M., Prasad, S. N., Hashmi, S., … Ramanathan, R. Dynamic interactions between peroxidase-mimic silver NanoZymes and chlorpyrifos-specific

aptamers enable highly-specific pesticide sensing in river water. *Analytica Chimica Acta*, **2019,** 1083, 157-165

2. Li, H., Liu, H., Zhang, J., Cheng, Y., Zhang, C., Fei, X., & Xian, Y. Platinum Nanoparticle Encapsulated Metal–Organic Frameworks for Colorimetric Measurement and Facile Removal of Mercury(II). *ACS Applied Materials & Interfaces*, **2017**, 9(46), 40716–40725.
3. Li, Z., Yang, X., Yang, Y., Tan, Y., He, Y., Liu, M., … Yuan, Q. (2017). Peroxidase-Mimicking Nanozyme with Enhanced Activity and High Stability Based on Metal-Support Interactions. *Chemistry - A European*, **2017**, 24(2), 409–415.
4. Zhang, Q., Zheng, S., Zhang, J. et al. Peroxidase Mimicking Activity of Palladium Nanocluster Altered by Heparin. *Catal Lett*, **2021**, 151, 2537–2546.
5. Feng, J., Huang, P., & Wu, F.-Y. Gold–platinum bimetallic nanoclusters with enhanced peroxidase-like activity and their integrated agarose hydrogel-based sensing platform for the colorimetric analysis of glucose levels in serum. *The Analyst*, **2017**, 142(21), 4106–4115.
